# Effects of light qualities on the growth and photosynthetic characteristics of *Sorbus tianschanica* Rupr. seedlings

**DOI:** 10.1371/journal.pone.0354303

**Published:** 2026-07-24

**Authors:** Xiong Lv, Shanchao Zhao, Hong Chen, Xin Zhao

**Affiliations:** 1 College of Forestry and Landscape Architecture, Xinjiang Agricultural University, Urumqi, Xinjiang, China; 2 Key Laboratory of Forestry Ecology and Industrial Technology in Arid Area, Xinjiang Education Department, Urumqi, Xinjiang, China; 3 Natural Forest Protection Center of Xinjiang Uygur Autonomous Region, Urumqi, Xinjiang, China; University of Agriculture Faisalabad, PAKISTAN

## Abstract

Light quality is an important environmental factor influencing plant growth and development, but its effects on functional traits and physiological processes in woody seedlings remain unclear. In this study, *Sorbus tianschanica* Rupr. seedlings were grown under four colored film treatments (red, blue, green, and white) to assess their effects on growth, leaf anatomy, chloroplast ultrastructure, photosynthetic performance, enzyme activities, and carbohydrate accumulation. Blue film significantly increased seedling height growth rate, whereas red film promoted ground diameter growth (*P* < 0.05). Stomatal aperture, size, and density were highest under blue film. Leaves under blue and red films were thicker and more compact, while those under green film were thinner and less structured. Chloroplasts in the blue treatment showed well-organized grana and stroma lamellae, whereas those under green film were disorganized. Photosynthetic pigment contents were higher under red, blue, and green films than under white film (*P* < 0.05). Net photosynthetic rate (*Pn*), stomatal conductance (*Gs*), and transpiration rate (*Tr*) were higher under blue and red films, and lowest under green film. Light response parameters, including maximum net photosynthetic rate (*Pn*_*max*_) and light saturation point (*LSP*), were also higher under blue and red treatments. Chlorophyll fluorescence parameters (*ΦPSII* and *qP*) and photosynthetic enzyme activities were highest under blue film. Blue film also increased carbohydrate accumulation, particularly in leaves and roots. Multilevel fuzzy comprehensive evaluation (MFCE) indicated that blue film had the highest overall performance. These results suggest that light quality affects seedling growth through changes in leaf structure, chloroplast organization, photosynthetic capacity, and carbon assimilation, with blue light providing the most favorable conditions for *Sorbus tianschanica* Rupr. seedlings.

## Introduction

Light is a key environmental factor regulating plant growth and development [1]. It influences plant morphology, photosynthetic performance, metabolic processes, fruit quality, and gene expression throughout the plant life cycle [1,2]. The light environment is primarily defined by light intensity, light quality, and photoperiod [2]. Among these, light quality refers to the spectral composition of light, which is perceived by plant photoreceptors and regulates downstream growth and developmental processes [3,4]. Previous studies have shown that red light promotes seedling height and stem diameter growth through photoreceptor-mediated signaling pathways [5,6]. In contrast, blue light regulates stomatal opening via cryptochrome-dependent responses, increases chlorophyll content, and enhances chlorophyll fluorescence parameters, thereby improving photosynthetic efficiency and promoting plant growth [7–10]. The combination of red and blue light can further improve light energy capture and utilization by modifying leaf morphology and anatomical structure, including increased leaf thickness and adjusted leaf area [11]. In addition, green light has been reported to enhance chlorophyll content and photosynthetic performance in some plant species [12,13]. However, the absence of blue light may impair normal morphological development and disrupt photosynthetic processes, ultimately inhibiting plant growth [14]. In practical applications, colored films with similar light transmittance are widely used to regulate light quality. These films selectively transmit specific wavelength ranges corresponding to their colors and offer advantages such as low cost and ease of application [12]. Therefore, modifying the light environment using colored films represents an effective strategy to enhance photosynthetic capacity, promote carbon assimilation, and improve plant growth [13].

*Sorbus tianschanica* Rupr. is native to the Tianshan Mountains at elevations of 1,400−2,000 m and is often associated with *Picea schrenkiana* var. *tianschanica*. Owing to its ornamental characteristics, including colorful flowers and fruits, this species has considerable potential for ecological restoration and urban greening [15,16]. However, the relatively slow growth of its seedlings limits large scale propagation and practical application. The seedling stage is a critical period determining subsequent growth and establishment [1], making the cultivation of high quality seedlings essential for afforestation and greening programs [17]. Light quality is a key environmental factor influencing plant growth, primarily through its effects on photosynthesis and carbon assimilation [1]. While these effects have been extensively studied in herbaceous crops, their application to woody plant seedlings remains limited [18,19]. In particular, there is a lack of systematic understanding of how different light qualities regulate photosynthetic processes, structural traits, and carbon allocation in *Sorbus tianschanica* Rupr. seedlings. We hypothesized that variation in light quality regulates seedling growth primarily through coordinated changes in photosynthetic capacity and carbon assimilation. To test this hypothesis, four types of PVC films (red, blue, green, and white) were used to establish distinct light quality environments. Seedling growth, stomatal characteristics, leaf anatomical structure, chloroplast ultrastructure, chlorophyll content, chlorophyll fluorescence parameters, photosynthetic enzyme activities, and sugar content were systematically measured. In addition, a multilevel fuzzy comprehensive evaluation (MFCE) model was applied to integrate multiple indicators and provide an overall assessment of seedling performance [20]. This study aimed to (i) clarify how different light qualities affect growth, photosynthetic characteristics, and carbon assimilation in *Sorbus tianschanica* Rupr. seedlings, and (ii) identify optimal light conditions to improve seedling quality. The results provide a theoretical basis for applying light regulation strategies to enhance the cultivation and utilization of woody plant species.

## Materials and methods

### Overview of the study site

The experiment was conducted in the nursery area of the Nanshan Internship Forestry Farm, Xinjiang Agricultural University (43°16′ N, 86°57′ E; 1,770 m above sea level). The site is located in a cold-temperate bioclimatic zone and is characterized by a relatively mild climate with moderate precipitation. The mean annual precipitation is approximately 600 mm, with about 60% occurring between May and August. The mean annual temperature is approximately 4 °C, and the site receives more than 1,300 hours of sunshine annually. The frost-free period is approximately 140 days. The soil is classified as typical gray-brown forest soil [16].

### Experimental materials and design

**Fig 1:**
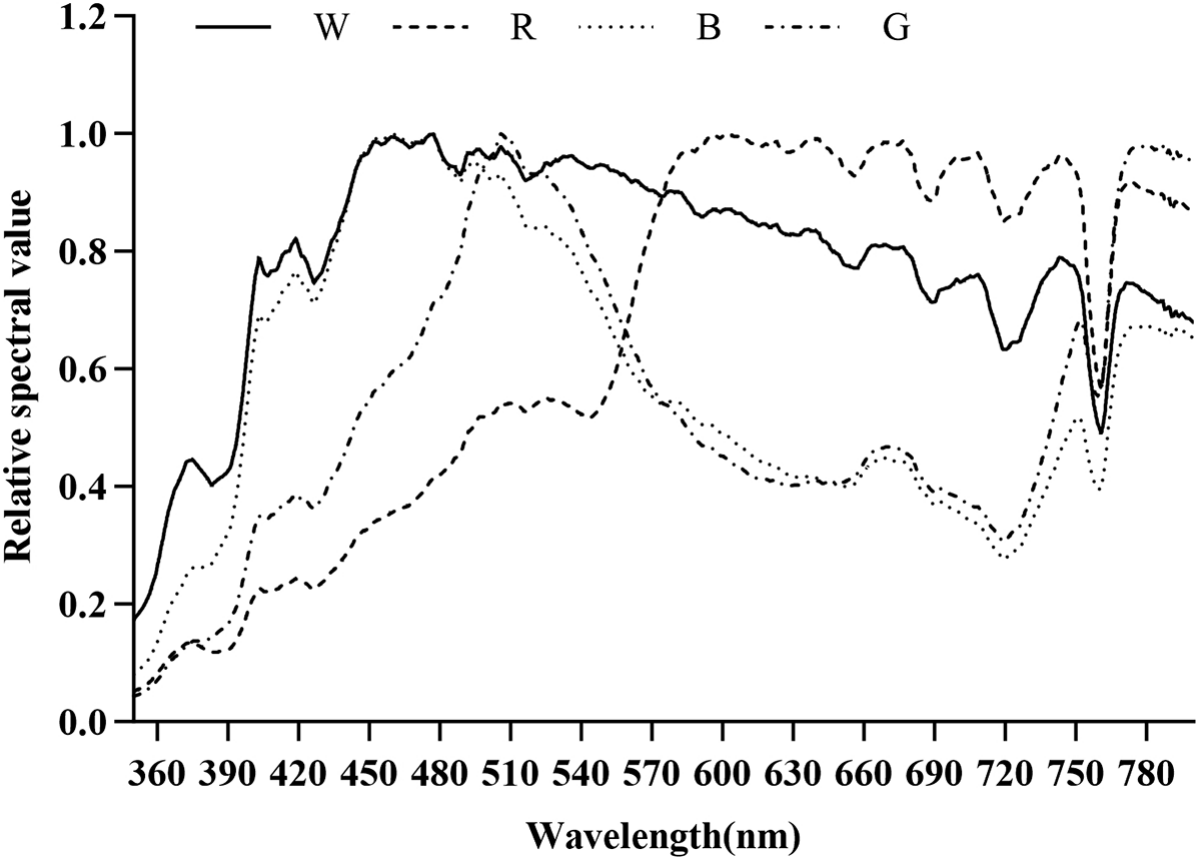
Relative spectral values of transmitted light under different color films.

Healthy, uniformly grown three-year-old *Sorbus tianschanica* Rupr. seedlings were used as experimental materials. In June 2025, seedbeds were covered with agricultural films of white (W), red (R), blue (B), and green (G) colors to establish different light quality conditions. The films, purchased from Lanzhou Golden Land Plastic Products Co., Ltd., had a thickness of 0.01 mm. Their spectral characteristics were measured using a PLA-30 plant light analyzer (Fig 1). Each film exhibited distinct spectral transmission peaks: red (602 nm), green (506 nm), and blue (459 nm), corresponding to their respective wavelength ranges.

The films were installed approximately 20 cm above the ground. Each treatment consisted of 30 seedlings with three replicates. All treatments were managed under identical conditions, including weeding, irrigation, and pest control. After 60 days, measurements were conducted for seedling growth, stomatal characteristics, leaf anatomical structure, chloroplast ultrastructure, photosynthetic pigment content, chlorophyll fluorescence parameters, photosynthetic enzyme activities, and sugar content.

### Measurement indicators and methods

#### Measurement of growth indicators.

Seedling height and ground diameter were measured before and after the treatment period. Seedling height was measured using a steel tape with an accuracy of 0.1 cm, and ground diameter was measured using a vernier caliper with an accuracy of 0.01 mm. Growth rates were calculated for each treatment based on the differences between initial and final measurements.


$$\begin{array}{c} \text{Growth rate}=\frac{\text{final measurement value }-\text{ initial measurement value}}{\text{initial measurement value}}\times \text{100}\% \end{array}$$ (1)

#### Leaf stomatal characterization.

Leaf stomatal traits were assessed using the nail polish impression method [21]. Stomatal length, width, circumference, area, and aperture were measured using a Motic-B5 optical microscope at 40 × magnification. Stomatal density, defined as the number of stomata per unit leaf area (mm^2^), was determined using a 10 × eyepiece.

#### Observations of leaf anatomy and chloroplast ultrastructure.

Leaf anatomical structures were examined using paraffin sections prepared according to Li et al. [22]. Observations were conducted using a Leica DM6000B microscope. Total leaf thickness, upper and lower epidermal thickness, palisade tissue thickness, and spongy tissue thickness were measured using Leica Application Suite micrometry software. Based on these measurements, the palisade-to-spongy tissue ratio (P/S), compactness of structure (CTR), and structure rarefaction (SR) were calculated to characterize leaf structure. For chloroplast ultrastructure analysis, fully expanded leaves of *Sorbus tianschanica* Rupr. seedlings were selected, avoiding major veins. Leaf tissues were cut into pieces smaller than 1 mm^2^ and immediately placed into vials. Samples were fixed in 2.5% glutaraldehyde, rinsed three times with 0.1 M phosphate buffer (pH = 7.2), and post-fixed in 2% osmium tetroxide for 2 h [23]. The samples were then dehydrated, embedded, and sectioned into ultrathin slices. Ultrathin sections were observed and imaged using a Hitachi transmission electron microscope.

#### Measurement of photosynthetic pigment content.

Photosynthetic pigments were extracted using a 95% ethanol extraction method following Yang et al. [24]. The absorbance of the extracts was measured using a UV spectrophotometer. Based on the absorbance values, the contents of chlorophyll a (*Chl a*), chlorophyll b (*Chl b*), carotenoids (*Car*), total chlorophyll (*Chl t*), and the chlorophyll a to b ratio (*Chl a/b*) were calculated.

#### Photosynthetic gas exchange parameters and light response curve determination.

The net photosynthetic rate (*Pn*), intercellular CO_2_ concentration (*Ci*), stomatal conductance (*Gs*), and transpiration rate (*Tr*) of fully expanded upper and middle leaves of *Sorbus tianschanica* Rupr. seedlings were measured using a LI-6800 portable photosynthesis system (LI-COR, USA). Measurements were conducted between 10:00 and 12:00 on clear days to ensure stable light conditions. Light response curves were determined under fourteen levels of photosynthetically active radiation (*PAR)*: 2000, 1800, 1500, 1200, 1000, 800, 600, 400, 200, 150, 100, 50, 20, and 0 μmol·m^-2^·s^-1^. The CO_2_ concentration in the leaf chamber was maintained at 400 μmol·mol^-1^. Based on these measurements, light response curves were generated for each treatment. Key parameters, including the maximum net photosynthetic rate (*Pn*_*max*_), apparent quantum efficiency (*AQY*), light compensation point (*LCP*), light saturation point (*LSP*), and dark respiration rate (*Rd*), were estimated by fitting the data to a non-rectangular hyperbolic model using regression analysis [25].

#### Determination of chlorophyll fluorescence parameters.

Chlorophyll fluorescence parameters were measured using an FMS-2 fluorometer. After light adaptation, minimum fluorescence of light-adapted leaves (F_O_′), maximum fluorescence (F_M_′), and steady-state fluorescence (F_S_) were recorded. Following dark adaptation, minimum fluorescence (F_O_) and maximum fluorescence (F_M_) were measured under dark-adapted conditions. Based on these values, the following parameters were calculated:

Maximum variable fluorescence (*Fv* = F_M_ - F_O_),
Actual photochemical efficiency of PSII (*ΦPSII* = (F_M_′ - F_S_)/F_M_′),
Maximum photochemical efficiency of PSII (*Fv/Fm* = (F_M_ - F_O_)/F_M_),
Non-photochemical quenching (*NPQ* = F_M_/F_M_′ - 1),
Photochemical quenching coefficient (*qP* = (F_M_′ - F_S_)/(F_M_′ - F_O_′),
Electron transport rate (*ETR = PAR*×0.5×*ΦPSII*×0.84).

#### Measurement of photosynthesis-related enzyme activities.

Leaf samples (1.5 g) were collected for each treatment, pooled, immediately frozen in liquid nitrogen, and stored for analysis. The samples were homogenized in 9 mL of phosphate buffer (pH = 7.4) at 4 °C. The homogenate was centrifuged at 5,000 rpm for 25 min, and the supernatant was collected for enzyme assays. The activities of ribulose-1,5-bisphosphate carboxylase/oxygenase (Rubisco), Rubisco activase (RCA), fructose-1,6-bisphosphatase (FBPase), glyceraldehyde-3-phosphate dehydrogenase (GAPDH), and ATP synthase were determined using ELISA kits (Shanghai Enzyme-linked Biotechnology Co., Ltd.). Absorbance was measured at 450 nm using a microplate reader, and enzyme activities were calculated based on the corresponding standard curves.

#### Measurement of sugar content.

Dried root, stem, and leaf samples were ground using a pulverizer and passed through a 100-mesh sieve. Soluble sugar contents were determined using the sulfuric acid-anthrone colorimetric method [26]. The concentrations of glucose, fructose, and sucrose were measured in roots, stems, and leaves for each treatment.

### Multilevel fuzzy comprehensive evaluation (MFCE) model

#### Construction of fuzzy evaluation factors and subfactor sets.

A total of 40 indicators were classified into nine categories to establish a comprehensive evaluation system for *Sorbus tianschanica* Rupr. seedlings. Seedling height growth rate and ground diameter growth rate were defined as morphological indicators (U1). Leaf stomatal length, width, area, perimeter, aperture, and density were grouped as stomatal traits (U2). Leaf thickness, upper epidermis thickness, palisade tissue thickness, spongy tissue thickness, lower epidermis thickness, the palisade-to-spongy tissue ratio (P/S), compactness of structure (CTR), and structure rarefaction (SR) were classified as leaf anatomical structure indicators (U3). Net photosynthetic rate (*Pn*), transpiration rate (*Tr*), intercellular CO_2_ concentration (*Ci*), and stomatal conductance (*Gs*) were categorized as gas exchange parameters (U4). Chlorophyll a (*Chl a*), chlorophyll b (*Chl b*), carotenoids (*Car*), total chlorophyll (*Chl t*), and the chlorophyll a/b ratio (*Chl a/b*) were defined as photosynthetic pigment indicators (U5). Chlorophyll fluorescence parameters, including actual photochemical efficiency of PSII (*ΦPSII*), photochemical quenching (*qP*), non-photochemical quenching (*NPQ*), electron transport rate (*ETR*), and maximum quantum efficiency of PSII (*Fv/Fm*), were grouped as fluorescence indicators (U6). Light response parameters, including apparent quantum efficiency (*AQY*), maximum net photosynthetic rate (*Pn*_*max*_), light saturation point (*LSP*), light compensation point (*LCP*), and dark respiration rate (*Rd*), were classified as light response indicators (U7). Photosynthetic enzyme activities, including ribulose-1,5-bisphosphate carboxylase/oxygenase (Rubisco), Rubisco activase (RCA), fructose-1,6-bisphosphatase (FBPase), glyceraldehyde-3-phosphate dehydrogenase (GAPDH), and ATP synthase, were categorized as enzyme activity indicators (U8). Finally, glucose, fructose, and sucrose contents in roots, stems, and leaves were grouped as carbohydrate indicators (U9).

#### Determination of MFCE factor weights.

The Analytic Hierarchy Process (AHP) [27] and the Entropy Weight (EW) method [28] were used to determine indicator weights. AHP was applied to calculate the subjective weights (W_AHP_) of the main factor layer, while the EW method was used to derive the objective weights (W_EW_) of the sub-factor layer. By integrating the subjective and objective weights, the final weight values were determined for each indicator category, including morphological indicators, stomatal traits, leaf anatomical structure indicators, gas exchange parameters, photosynthetic pigment indicators, fluorescence parameters, light response parameters, photosynthetic enzyme activities, and glucose, fructose, and sucrose contents.

#### MFCE index calculation.

Fuzzy comprehensive evaluation was performed on the weighted indicators to obtain a composite evaluation index [29].

#### Statistics and analysis of data.

Experimental data were organized using Excel 2019 and visualized with GraphPad Prism. Yaahp 10.01 was used to construct the comprehensive evaluation hierarchy model for *Sorbus tianschanica* Rupr. seedlings and to calculate the weights of each indicator. Statistical analyses were conducted using SPSS 23.0. Differences among treatments were assessed using Duncan’s multiple range test.

## Results and analysis

### Effect of light quality on seedling height growth rate and diameter growth rate of *Sorbus tianschanica* Rupr. seedlings

**Fig 2:**
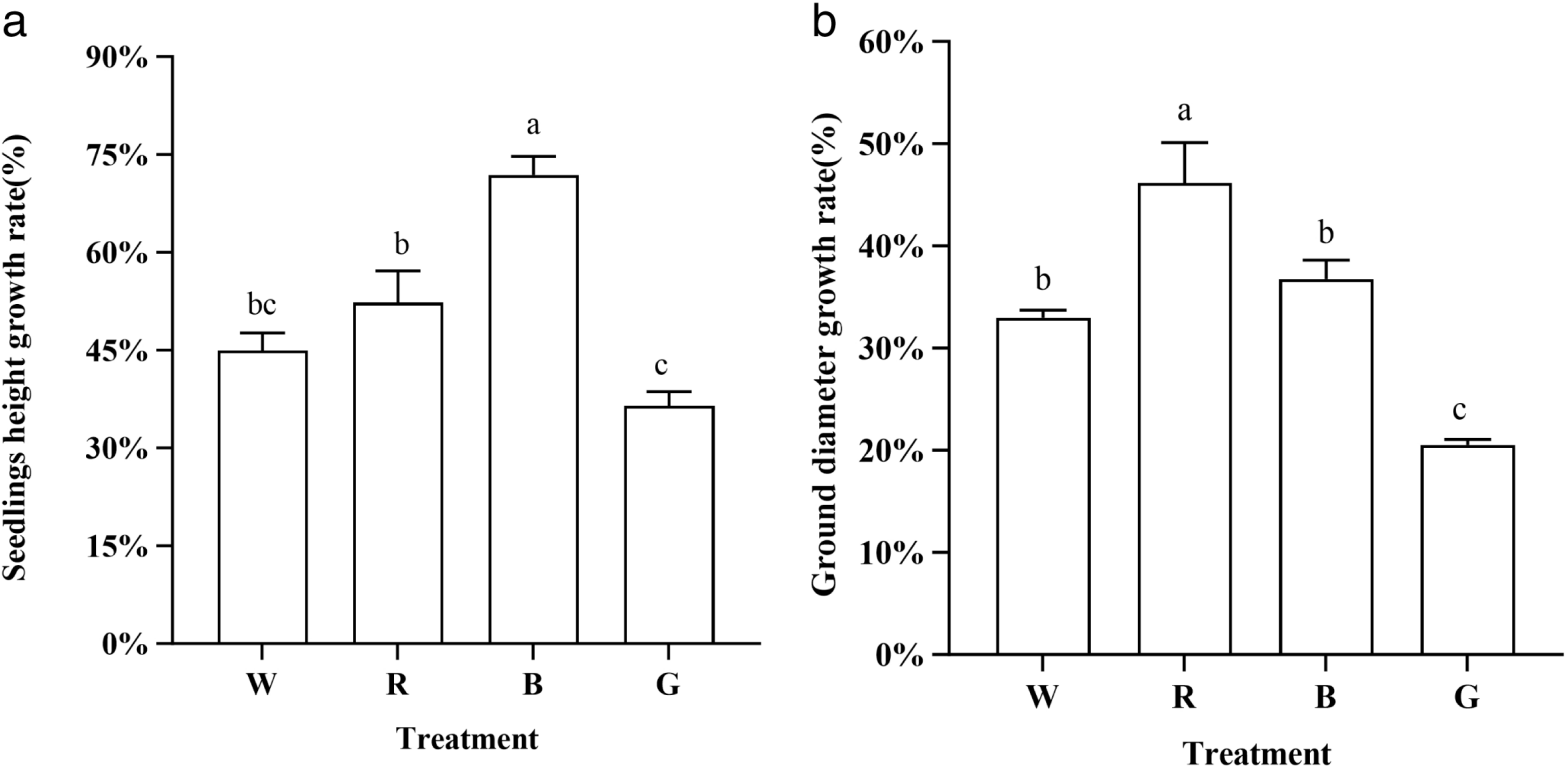
Effect of light quality on seedling height growth rate and ground diameter growth rate of *Sorbus tianschanica* Rupr. seedlings. (a) Seedling height growth rate; (b) Ground diameter growth rate. Different letters in the figure indicate significant differences (*P* < 0.05). Same as below.

As shown in Fig 2, the highest seedling height growth rate was observed under the blue film treatment (71.89%), which was significantly higher than those under the red, green, and white film treatments (*P* < 0.05) (Fig 2a). No significant differences in height growth rate were detected among the red, green, and white treatments (*P* > 0.05). For ground diameter, the red film treatment resulted in the highest growth rate (46.19%), which was significantly greater than those under the blue, green, and white film treatments (*P* < 0.05) (Fig 2b). No significant difference in diameter growth rate was observed between the blue and white treatments (*P* > 0.05).

### Effect of light quality on stomatal characteristics of *Sorbus tianschanica* Rupr. seedlings

As shown in Table 1, stomatal length of *Sorbus tianschanica* Rupr. seedlings under red and blue film treatments did not differ significantly from that under the white film treatment (*P* > 0.05). However, stomatal length was lowest under the green film treatment (27.0 μm), which was significantly lower than that under the blue and white treatments (*P* < 0.05). Stomatal width and area were highest under the blue film treatment, reaching 22.6 μm and 575.1 μm^2^, respectively. These values were significantly greater than those under the green film treatment (*P* < 0.05), but did not differ significantly from those under the red and white treatments (*P* > 0.05). Stomatal perimeter under the blue film treatment was significantly greater than that under the red and green treatments (*P* < 0.05), but showed no significant difference compared with the white treatment (*P* > 0.05). Stomatal aperture was also highest under the blue film treatment (9.6 μm), which was significantly greater than that under all other treatments (*P* < 0.05). In contrast, stomatal density was lowest under the red film treatment (24.5 per/mm^2^), which was significantly lower than that under the blue treatment (*P* < 0.05), but did not differ significantly from the white and green treatments (*P* > 0.05).

**Table 1:** Effects of light qualities on stomatal characteristics of *Sorbus tianschanica* Rupr. seedlings.

Treatment^*^	Length/μm	Width/μm	Square/μm^2^	Circumference/μm	Aperture/μm	SD(per/mm^2^)
**W**	30.8 ± 0.65a	22.3 ± 0.34a	522.8 ± 21.97ab	83.7 ± 1.76ab	7.6 ± 0.31b	27.9 ± 2.41ab
**R**	29.6 ± 1.28ab	21.2 ± 0.85ab	510.3 ± 31.52ab	81.5 ± 1.96bc	7.9 ± 0.84b	24.5 ± 1.56b
**B**	31.5 ± 0.99a	22.6 ± 0.24a	575.1 ± 14.82a	88.0 ± 1.24a	9.6 ± 0.27a	32.0 ± 1.35a
**G**	27.0 ± 0.82b	20.5 ± 0.39b	470.1 ± 10.99b	78.8 ± 0.70c	7.5 ± 0.14b	26.4 ± 1.71ab

*Different letters in the table indicate significant differences (*P* < 0.05). Same as below.

### Effect of light quality on the anatomical structure of leaves of *Sorbus tianschanica* Rupr. seedlings

As shown in Table 2, leaf thickness, upper epidermal thickness, palisade tissue thickness, palisade-to-spongy tissue ratio (P/S), and compactness of structure (CTR) of *Sorbus tianschanica* Rupr. seedlings were highest under the blue film treatment, with values of 233.87 μm, 30.67 μm, 120.78 μm, 1.88, and 0.52, respectively. These values were significantly greater than those under the white and green film treatments (*P* < 0.05). Lower epidermal thickness was highest under the red film treatment (13.37 μm), which was significantly greater than that under the white, blue, and green treatments (*P* < 0.05). However, no significant differences were observed among the red, blue, and white treatments (*P* > 0.05). Spongy tissue thickness and structure rarefaction (SR) were highest under the green film treatment, reaching 102.78 μm and 0.51, respectively. These values were significantly higher than those under the white, red, and blue treatments (*P* < 0.05). No significant differences were observed between the white and red treatments for these parameters (*P* > 0.05).

**Table 2:** Effect of light qualities on anatomical structure of leaves of *Sorbus tianschanica* Rupr. seedlings.

Treatment	W	R	B	G
**Leaf thickness/μm**	209.40 ± 5.70bc	227.85 ± 5.14ab	233.87 ± 6.88a	202.84 ± 3.48c
**Upper epidermal thickness/μm**	17.90 ± 1.44c	24.22 ± 0.85b	30.67 ± 0.74a	23.79 ± 0.88b
**Palisade tissue thickness/μm**	93.96 ± 1.76c	110.12 ± 1.37b	120.78 ± 3.28a	76.52 ± 2.28d
**Spongy tissue thickness/μm**	82.03 ± 3.35b	84.25 ± 2.82b	65.03 ± 4.51c	102.78 ± 1.81a
**Lower epidermal thickness/μm**	12.6 ± 0.69ab	13.37 ± 0.27a	13.19 ± 0.57a	10.95 ± 0.25b
**P/S**	1.15 ± 0.05b	1.31 ± 0.05b	1.88 ± 0.09a	0.75 ± 0.03c
**CTR**	0.45 ± 0.01b	0.48 ± 0.01ab	0.52 ± 0.01a	0.38 ± 0.01c
**SR**	0.39 ± 0.01b	0.37 ± 0.01b	0.28 ± 0.01c	0.51 ± 0.01a

### Effect of light quality on chloroplast ultrastructure in *Sorbus tianschanica* Rupr. seedlings

**Fig 3:**
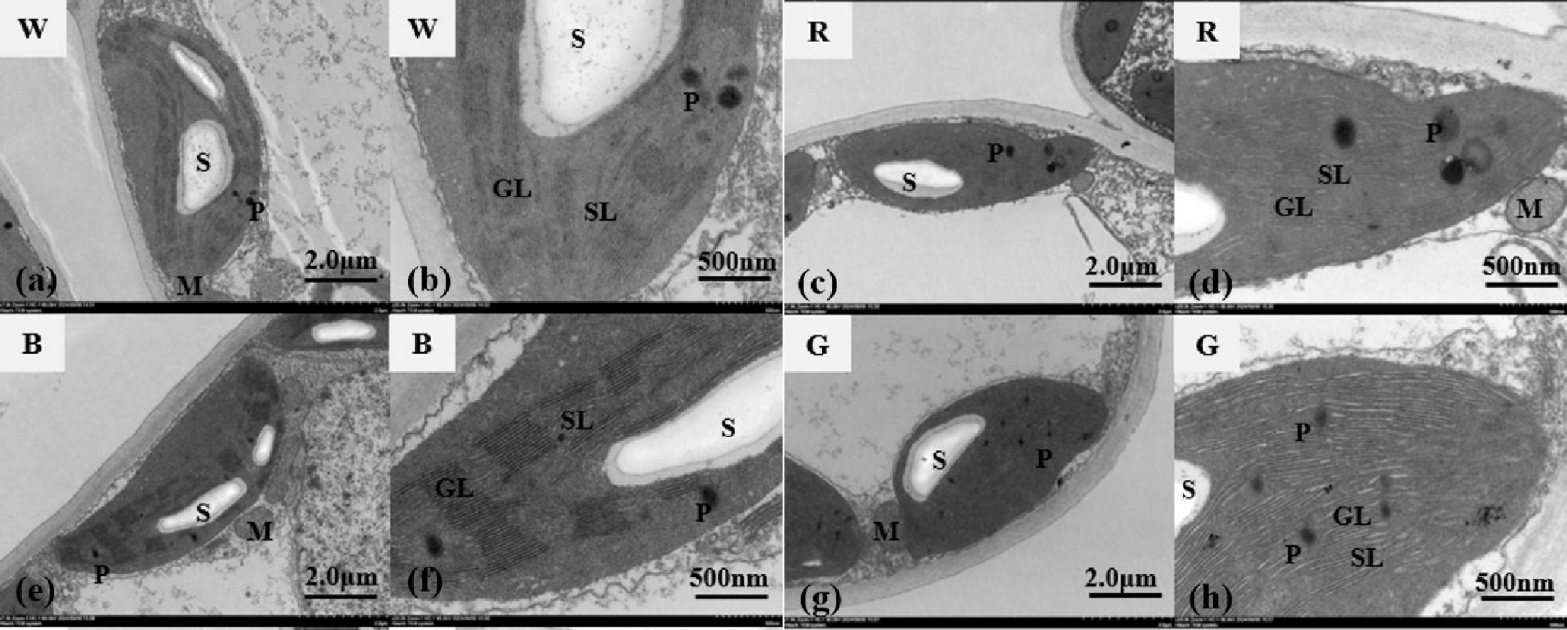
Effect of light quality on the ultrastructure of chloroplasts of *Sorbus tianschanica* Rupr. seedlings. M: Mitochondria; S: Starch grain; GL: Grana lamella; SL: Stroma lamella; P: Plastoglobulus.

As shown in Fig 3, chloroplasts in *Sorbus tianschanica* Rupr. leaves exhibited distinct structural differences under different light quality treatments. Under the white film treatment (Fig 3a and 3b), chloroplasts displayed well-organized grana and stroma lamellae, with numerous grana stacks, enlarged starch granules, and a few osmiophilic granules. Under the red film treatment (Fig 3c and 3d), chloroplast structure remained well organized, with clearly defined grana and stroma lamellae. Chloroplasts contained enlarged starch granules, and the grana stacks appeared more densely arranged compared with the white treatment. In addition, osmiophilic granules were more prominent. Under the blue film treatment (Fig 3e and 3f), chloroplasts showed a highly organized structure, with tightly stacked grana and well-developed lamellar systems. Compared with the white treatment, grana stacking was denser and more compact, while starch granules were relatively smaller and the number of osmiophilic granules was reduced. In contrast, chloroplasts under the green film treatment (Fig 3g and 3h) exhibited a disorganized ultrastructure, characterized by loosely arranged and poorly defined grana and stroma lamellae. These chloroplasts contained enlarged starch granules and a high number of osmiophilic granules.

### Effect of light quality on photosynthetic pigment content of *Sorbus tianschanica* Rupr. seedlings

**Fig 4:**
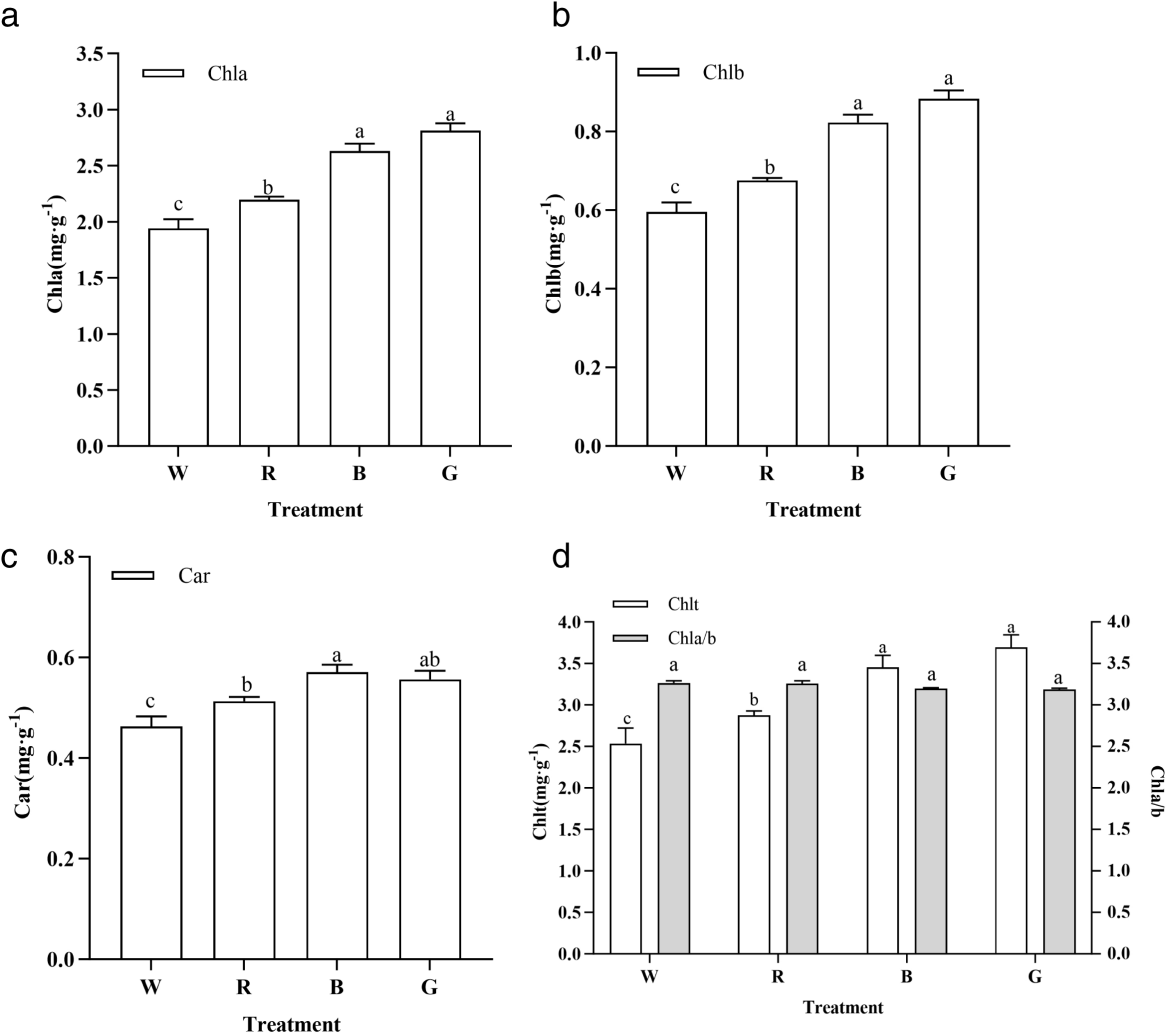
Effect of light quality on photosynthetic pigment content of *Sorbus tianschanica* Rupr. seedlings. (a) Chlorophyll a (Chl a); (b) Chlorophyll b (Chl b); (c) Carotenoids (Car); (d) Total chlorophyll (Chl t) and chlorophyll a/b ratio (Chl a/b).

As shown in Fig 4, the contents of chlorophyll a (*Chl a*) (Fig 4a), chlorophyll b (*Chl b*) (Fig 4b), carotenoids (*Car*) (Fig 4c), and total chlorophyll (*Chl t*) (Fig 4d) in *Sorbus tianschanica* Rupr. leaves were significantly higher under the red, blue, and green film treatments than under the white film treatment (*P* < 0.05). In contrast, the chlorophyll a/b ratio (*Chl a/b*) (Fig 4d) did not differ significantly among treatments (*P* > 0.05). Among the treatments, the highest *Chl a* (Fig 4a), *Chl b* (Fig 4b), and *Chl t* (Fig 4d) contents were observed under the green film treatment (2.81, 0.88, and 3.69 mg·g^-1^, respectively), which did not differ significantly from those under the blue film treatment (*P* > 0.05). The *Car* content was highest under the blue film treatment (0.57 mg·g^-1^), but did not differ significantly from that under the green film treatment (*P* > 0.05) (Fig 4c). Both blue and green treatments showed significantly higher pigment contents than the red treatment (*P* < 0.05).

### Effect of light quality on photosynthetic parameters of *Sorbus tianschanica* Rupr. seedlings

**Fig 5:**
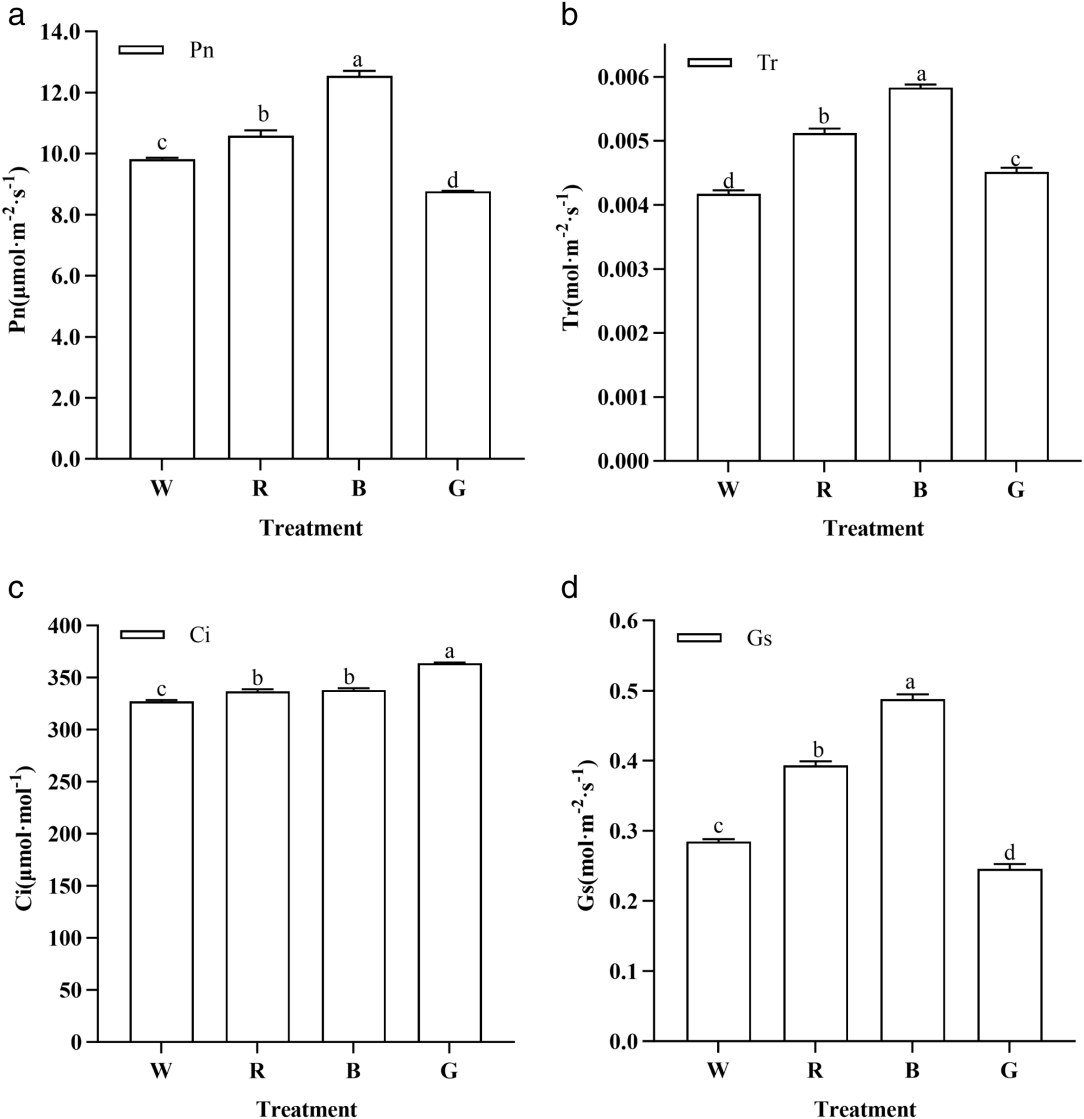
Effects of light qualities on photosynthetic parameters of *Sorbus tianschanica* Rupr. seedlings. (a) Net photosynthetic rate (Pn); (b) Transpiration rate (Tr); (c) Intercellular CO_2_ concentration (Ci); (d) Stomatal conductance (Gs).

As shown in Fig 5, the net photosynthetic rate (*Pn*) of *Sorbus tianschanica* Rupr. leaves was highest under the blue film treatment (12.55 μmol·m^-2^·s^-1^), which was significantly greater than that under the red, green, and white treatments (*P* < 0.05) (Fig 5a). The transpiration rate (*Tr*) was significantly higher under the red, blue, and green treatments than under the white treatment (*P* < 0.05) (Fig 5b). Among these, *Tr* was highest under the blue film treatment (0.0058 mol·m^-2^·s^⁻¹^), which was significantly greater than that under the red and green treatments (*P* < 0.05) (Fig 5b). Intercellular CO_2_ concentration (*Ci*) was significantly higher under the red, blue, and green treatments than under the white treatment (*P* < 0.05) (Fig 5c). No significant difference in *Ci* was observed between the red and blue treatments (*P* > 0.05), while the highest value was recorded under the green treatment (363.70 μmol·mol^-1^) (Fig 5c). Stomatal conductance (*Gs*) was highest under the blue film treatment (0.49 mol·m^-2^·s^-1^), which was significantly greater than that under the red, green, and white treatments (*P* < 0.05) (Fig 5d).

### Effect of light quality on light response of *Sorbus tianschanica* Rupr. seedlings

**Fig 6:**
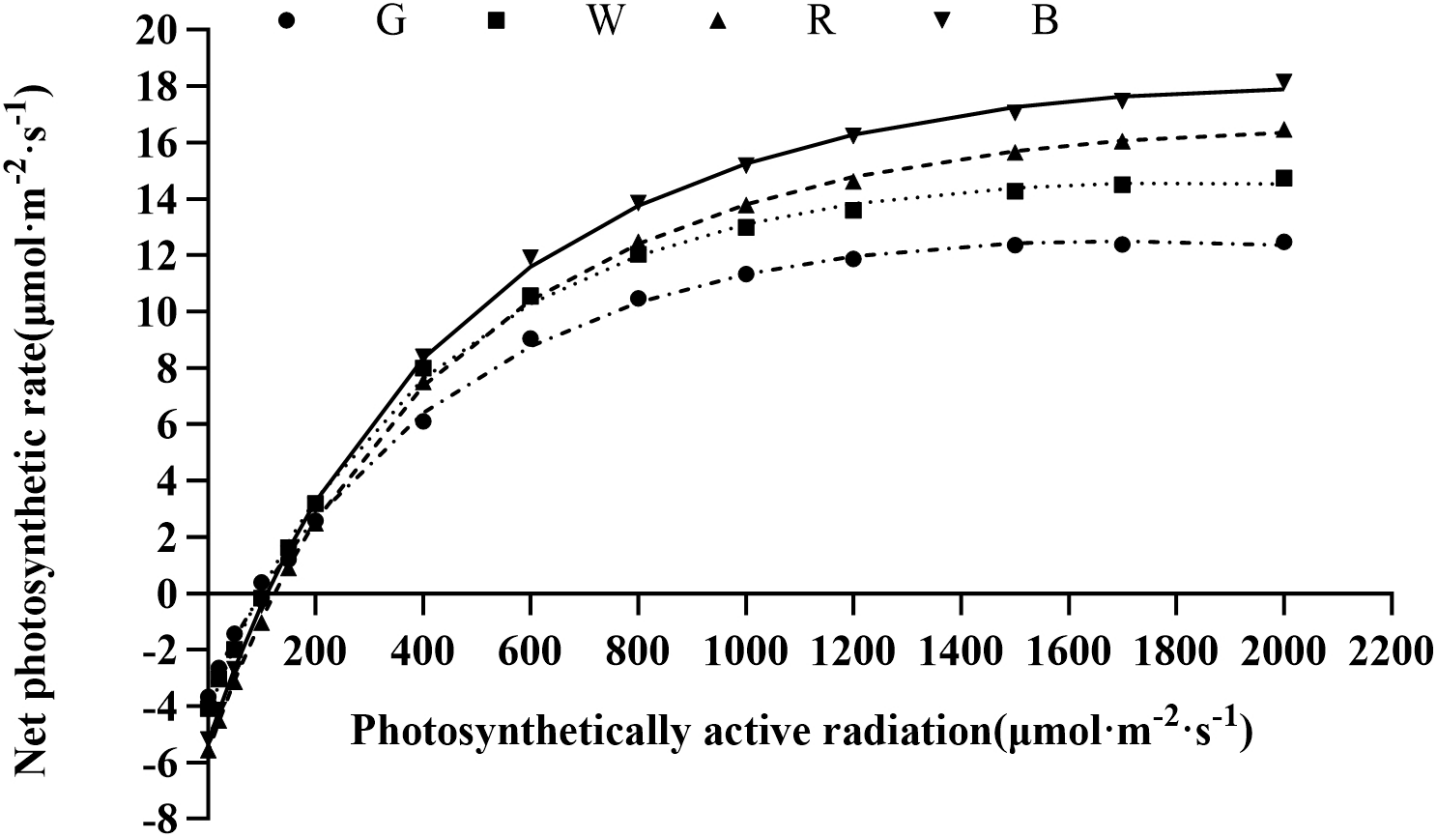
Effects of light qualities on the light response curves of *Sorbus tianschanica* Rupr. seedlings.

As shown in Fig 6, the light response curves of *Sorbus tianschanica* Rupr. leaves under different film treatments exhibited similar overall patterns. The net photosynthetic rate (*Pn*) increased rapidly with increasing photosynthetically active radiation (*PAR*) at low light levels (0-400 μmol·m^-2^·s^-1^), followed by a gradual increase and eventual stabilization at higher PAR (>800 μmol·m^-2^·s^-1^), indicating a typical light saturation response. However, clear differences in *Pn* were observed among treatments. Across the entire *PAR* range, the blue film treatment showed the highest *Pn*, followed by the red and white treatments, while the green treatment consistently exhibited the lowest values. At high *PAR* levels, *Pn* under the blue treatment reached the highest plateau, indicating a greater photosynthetic capacity and higher light utilization efficiency compared with the other treatments.

As shown in Table 3, the apparent quantum efficiency (*AQY*) of leaves under the green film treatment was significantly lower than that under the red, blue, and white treatments (*P* < 0.05), whereas no significant differences were observed among the red, blue, and white treatments (*P* > 0.05). The maximum net photosynthetic rate (*Pn*_*max*_) was significantly higher under the blue, red, and white treatments than under the green treatment (*P* < 0.05). Among these, the highest *Pn*_*max*_ was observed under the blue film treatment (17.93 μmol·m^-2^·s^-1^), while the lowest value occurred under the green treatment (12.97 μmol·m^-2^·s^-1^). Differences in *Pn*_*max*_ among all treatments were statistically significant (*P* < 0.05). The light saturation point (*LSP*) was highest under the red film treatment (2225.21 μmol·m^-2^·s^-1^), which did not differ significantly from the blue treatment (*P* > 0.05), but was significantly higher than the white and green treatments (*P* < 0.05). Similarly, the light compensation point (*LCP*) was highest under the red treatment (124.51 μmol·m^-2^·s^-1^), with no significant difference compared to the blue treatment (*P* > 0.05), but significantly higher than the white and green treatments (*P* < 0.05). No significant differences in *LCP* were observed among the blue, green, and white treatments (*P* > 0.05). Dark respiration rate (*Rd*) differed significantly among treatments (*P* < 0.05). *Rd* was higher under the red and blue treatments than under the white and green treatments (*P* < 0.05), while the lowest value was observed under the green treatment.

**Table 3:** Effects of light qualities on light response parameters of *Sorbus tianschanica* Rupr. seedlings.

Treatment	*AQY* (mol·mol^-1^)	*Pn*_*max*_ (μmol·m^-2^·s^-1^)	*LSP* (μmol·m^-2^·s^-1^)	*LCP* (μmol·m^-2^·s^-1^)	*Rd* (μmol·m^-2^·s^-1^)
**W**	0.053 ± 0.001a	14.652 ± 0.048c	1739.028 ± 50.731b	97.028 ± 0.088b	4.359 ± 0.068c
**R**	0.056 ± 0.005a	16.398 ± 0.091b	2225.205 ± 22.045a	124.505 ± 9.818a	5.635 ± 0.013a
**B**	0.057 ± 0.001a	17.929 ± 0.222a	2172.657 ± 59.294a	109.706 ± 0.920ab	5.286 ± 0.024b
**G**	0.042 ± 0.001b	12.972 ± 0.277d	1638.942 ± 37.677b	97.180 ± 1.233b	3.591 ± 0.071d

### Effect of light quality on chlorophyll fluorescence parameters of *Sorbus tianschanica* Rupr. seedlings

As shown in Table 4, the maximum quantum efficiency of PSII (*Fv/Fm*) was similar among treatments, with values of 0.83, 0.86, and 0.86 under the red, blue, and green film treatments, respectively, indicating no substantial differences in PSII photochemical efficiency.The actual photochemical efficiency (*ΦPSII*) was highest under the blue film treatment (0.59), which was significantly greater than that under the white, red, and green treatments (*P* < 0.05). No significant differences in *ΦPSII* were observed among the red, green, and white treatments (*P* > 0.05). Similarly, the photochemical quenching coefficient (*qP*) was highest under the blue treatment (0.81), significantly exceeding the other treatments (*P* < 0.05). In contrast, non-photochemical quenching (*NPQ*) did not differ significantly among treatments (*P* > 0.05). The electron transport rate (*ETR*) showed significant variation (*P* < 0.05), with the highest value observed under the white film treatment (6.55), which was significantly greater than those under the red, blue, and green treatments. The lowest *ETR* was recorded under the red treatment (2.56).

**Table 4:** Effects of light qualities on chlorophyll fluorescence parameters of *Sorbus tianschanica* Rupr. seedlings.

Treatment	*Fv/Fm*	*ΦPSII*	*qP*	*NPQ*	*ETR*
**W**	0.81 ± 0.01c	0.54 ± 0.01b	0.78 ± 0.01b	1.10 ± 0.09a	6.55 ± 0.17a
**R**	0.83 ± 0.01b	0.54 ± 0.01b	0.77 ± 0.01b	1.15 ± 0.09a	2.56 ± 0.16d
**B**	0.86 ± 0.01a	0.59 ± 0.01a	0.81 ± 0.01a	0.97 ± 0.07a	4.02 ± 0.15c
**G**	0.86 ± 0.00a	0.53 ± 0.01b	0.72 ± 0.01c	0.95 ± 0.09a	5.30 ± 0.19b

### Effect of light quality on photosynthetic enzyme activities of *Sorbus tianschanica* Rupr. seedlings

**Fig 7:**
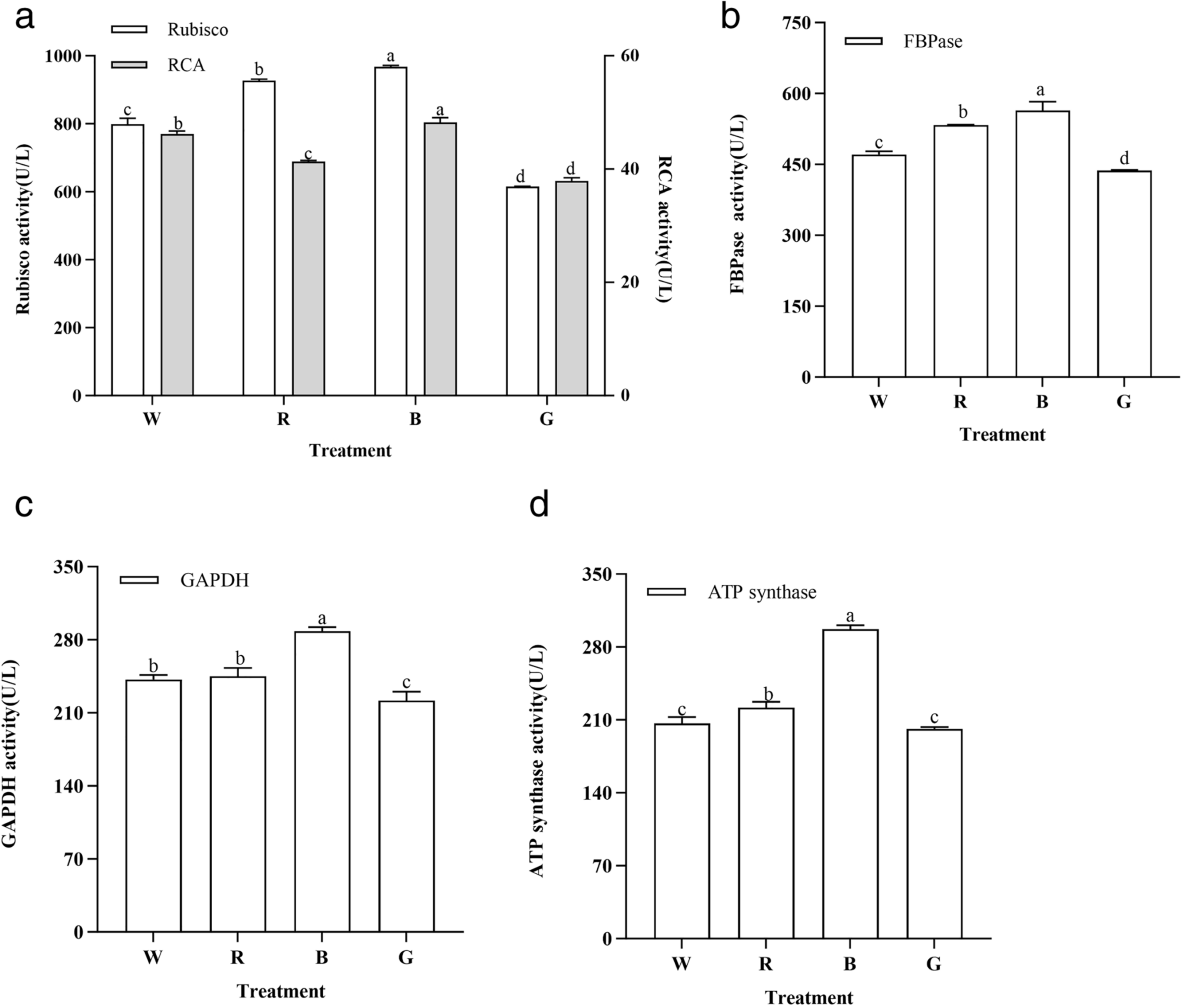
Effects of light qualities on photosynthetic enzyme activities of *Sorbus tianschanica* Rupr. seedlings. (a) Activities of ribulose-1,5-bisphosphate carboxylase/oxygenase (Rubisco) and Rubisco activase (RCA); (b) Activity of fructose-1,6-bisphosphatase (FBPase); (c) Activity of glyceraldehyde-3-phosphate dehydrogenase (GAPDH); (d) Activity of ATP synthase.

As shown in Fig 7, the activities of ribulose-1,5-bisphosphate carboxylase/oxygenase (Rubisco) (Fig 7a), rubisco activase (RCA) (Fig 7a), and fructose-1,6-bisphosphatase (FBPase) (Fig 7b) were highest under the blue film treatment, reaching 967.98, 48.24, and 563.98 U/L, respectively. These values were significantly greater than those under the white, red, and green treatments (*P* < 0.05), and all three enzymes showed significant variation among treatments (*P* < 0.05). Glyceraldehyde-3-phosphate dehydrogenase (GAPDH) activity was also highest under the blue film treatment (288.30 U/L), which was significantly higher than that under the other treatments (*P* < 0.05) (Fig 7c). No significant difference in GAPDH activity was observed between the white and red treatments (*P* > 0.05) (Fig 7c). Similarly, ATP synthase activity was highest under the blue film treatment (297.15 U/L), significantly exceeding the other treatments (*P* < 0.05) (Fig 7d). However, no significant difference was observed between the white and green treatments (*P* > 0.05).

### Effect of light quality on sugar content in *Sorbus tianschanica* Rupr. seedlings

**Fig 8:**
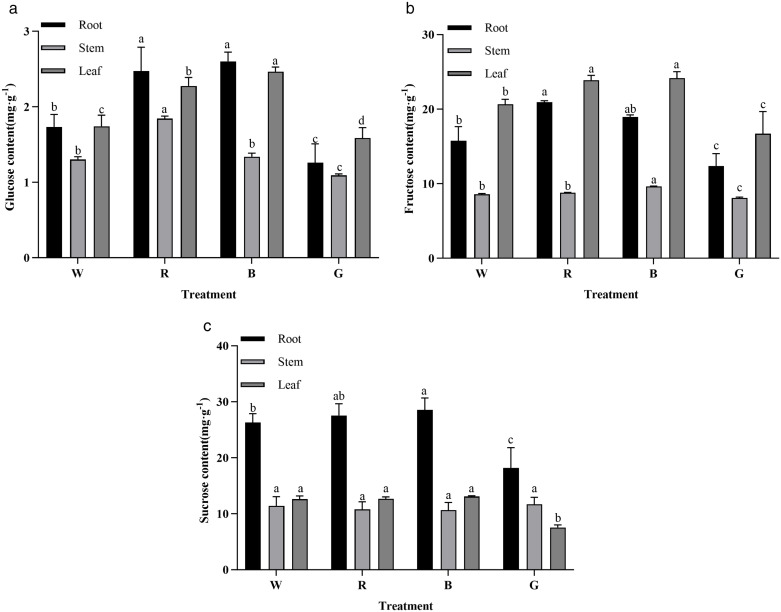
Effects of light qualities on sugar content of *Sorbus tianschanica* Rupr. seedlings. (a) Glucose content; (b) Fructose content; (c) Sucrose content.

As shown in Fig 8, sugar contents varied among treatments and plant organs. For glucose (Fig 8a), the highest concentrations in roots and leaves were observed under the blue film treatment (2.60 and 2.46 mg·g^-1^, respectively), while stem glucose content was highest under the red film treatment (1.84 mg·g^-1^). Root glucose content under the blue treatment was significantly higher than that under the white and green treatments (*P* < 0.05), but did not differ from the red treatment (*P* > 0.05) (Fig 8a). Stem glucose content under the red treatment was significantly higher than that under all other treatments (*P* < 0.05) (Fig 8a). Leaf glucose content was highest under the blue treatment, and significant differences were observed among all treatments (*P* < 0.05) (Fig 8a). For fructose (Fig 8b), the highest concentrations in stems and leaves were observed under the blue treatment (9.63 and 24.18 mg·g^-1^, respectively), whereas root fructose content was highest under the red treatment (20.94 mg·g^-1^). Root fructose content under the red treatment was significantly higher than that under the white and green treatments (*P* < 0.05), but did not differ from the blue treatment (*P* > 0.05) (Fig 8b). Stem fructose content under the blue treatment was significantly higher than that under all other treatments (*P* < 0.05) (Fig 8b). Leaf fructose content under the blue treatment was significantly higher than that under the white and green treatments (*P* < 0.05), but did not differ from the red treatment (*P* > 0.05) (Fig 8b). For sucrose (Fig 8c), the highest concentrations in roots and leaves were observed under the blue treatment (28.54 and 13.13 mg·g^-1^, respectively), while stem sucrose content was highest under the green treatment (11.69 mg·g^-1^). Root sucrose content under the blue treatment was significantly higher than that under the white and green treatments (*P* < 0.05), but did not differ from the red treatment (*P* > 0.05) (Fig 8c). Stem sucrose content did not differ significantly among treatments (*P* > 0.05) (Fig 8c). Leaf sucrose content under the blue treatment was significantly higher than that under the green treatment (*P* < 0.05), but did not differ from the white or red treatments (*P* > 0.05) (Fig 8c).

### Comprehensive evaluation of *Sorbus tianschanica* Rupr. seedlings based on the MFCE model

The multilevel fuzzy comprehensive evaluation (MFCE) model was used to assess overall seedling performance under different treatments (Tables 5 and 6). The comprehensive growth index (CGI) was highest under the blue film treatment (0.4243), followed by the red (0.2855), white (0.1952), and green (0.0949) treatments. These results indicate that the blue film treatment provided the most favorable conditions for the growth and physiological performance of *Sorbus tianschanica* Rupr. seedlings.

**Table 5:** Subjective and objective weights based on AHP and EW.

Factor	Subfactor	W_AHP_	W_EW_	Factor	Subfactor	W_AHP_	W_EW_
**U1**	Seedling height growth rate	0.036	0.022	**U6**	*ΦPSII*	0.075	0.044
	Ground diameter growth rate		0.016		*qP*		0.016
**U2**	Pore length	0.025	0.015		*NPQ*		0.021
	Pore width		0.019		*ETR*		0.018
	Pore area		0.018		*Fv/Fm*		0.017
	Pore circumference		0.020	**U7**	*AQY*	0.051	0.015
	Pore opening		0.038		*Pn*_*max*_		0.018
	Pore density		0.022		*LSP*		0.023
**U3**	Leaf thickness	0.109	0.021		*LCP*		0.036
	Upper epidermal thickness		0.017		*Rd*		0.018
	Palisade tissue thickness		0.017	**U8**	Rubisco	0.309	0.016
	Spongy tissue thickness		0.017		RCA		0.018
	Lower epidermal thickness		0.015		FBPase		0.020
	Palisade-to-spongy tissue ratio		0.019		GAPDH		0.022
	CTR		0.016		ATP synthase		0.037
	SR		0.016	**U9**	RootGlu	0.154	0.018
**U4**	*Pn*	0.224	0.021		RootFru		0.017
	*Tr*		0.022		RootSuc		0.014
	*Ci*		0.015		StemGlu		0.023
	*Gs*		0.024		StemFru		0.020
**U5**	*Chl a*	0.018	0.019		StemSuc		0.025
	*Chl b*		0.020		LeafGlu		0.023
	*Car*		0.016		LeafFru		0.016
	*Chl t*		0.019		LeafSuc		0.014
	*Chl a/b*		0.024				

**Table 6:** Final comprehensive growth index of *Sorbus tianschanica* Rupr. seedlings.

Treatment	CGI	Rank
**W**	0.1952	3
**R**	0.2855	2
**B**	0.4243	1
**G**	0.0949	4

## Discussion

Light quality is a key regulator of plant morphogenesis and growth, as plants perceive and respond to specific wavelengths through photoreceptor-mediated pathways [1]. Seedling height and stem diameter are sensitive indicators of growth responses to light conditions [2]. In this study, blue film significantly increased the height growth rate of *Sorbus tianschanica* Rupr. seedlings, consistent with results reported for *Populus szechuanica* var. *tibetica* [30] and *Cucumis sativus* [31]. This response may be associated with blue light regulation of gibberellin (GA) signaling and DELLA protein dynamics, which promote stem elongation [32,33]. In contrast, red film enhanced ground diameter growth, consistent with findings in *Solanum lycopersicum* [34]. This effect is likely related to changes in the red-to-far-red ratio (R/FR), which activate phytochrome B (phyB)-mediated signaling pathways, regulate phytochrome-interacting factors (PIFs), and promote lateral cell expansion [35].

Stomatal traits are critical for gas exchange and photosynthesis [36]. Blue light has been shown to promote stomatal opening in *Cucumis melo* and *Arabidopsis thaliana* [37,38], increase stomatal area in *Abelmoschus manihot* [39] and *Brassica rapa* [40], and enhance stomatal density in *Solanum* *lycopersicum* [34], although species-specific responses exist, as observed in *Dendrobium officinale* [41]. In this study, stomatal size, aperture, and density were all higher under blue film, likely due to activation of plasma membrane H ⁺ -ATPase [42]. These changes enhance CO_2_ diffusion and support higher photosynthetic activity. Leaf anatomical structure is closely linked to photosynthetic capacity [36,43]. Leaves under blue and red films were thicker and more compact, whereas those under green film were thinner and loosely arranged. Similar responses have been reported in *Gossypium hirsutum* [44] and *Solanum lycopersicum* [45]. Increased palisade tissue development under blue and red light likely enhances chloroplast density and photosynthetic efficiency [44]. In contrast, green film promoted spongy tissue development and structural looseness, which may increase internal light scattering but reduce the density of photosynthetic organelles per unit area. This response may reflect shade-avoidance mechanisms induced by green light through altered photoreceptor signaling and hormone regulation [46]. Chloroplast ultrastructure is strongly associated with photosynthetic performance [43]. In this study, red and blue films promoted well-organized grana and stroma lamellae, consistent with findings in *Vitis vinifera* [47], *Cucumis sativus* [48], and *Malus domestica* [49]. Red light likely regulates chloroplast development via phytochrome-mediated pathways [50], whereas blue light enhances the synthesis of photosynthetic membrane proteins through cryptochrome and phototropin signaling [51]. In contrast, green film resulted in disorganized chloroplast structure, with enlarged starch granules and increased osmiophilic granules, indicating impaired photosynthetic function [52].

Photosynthetic pigment content reflects the capacity for light absorption and energy conversion [53]. In this study, chlorophyll and carotenoid contents were higher under red, blue, and green films, with the highest values under blue and green treatments. Similar trends have been reported in *Vaccinium* spp. [12], *Populus szechuanica* var. *tibetica* [30], *Prunus persica* [54], and *Ginkgo biloba* [55]. Blue light may enhance chlorophyll biosynthesis through cryptochrome-mediated regulation [56], while increased pigment content under green light may represent a compensatory response to reduced effective light absorption [57]. However, species-specific variation exists, as *Pisum sativum* shows maximum pigment accumulation under red light [58]. Photosynthetic performance was significantly influenced by light quality. Blue and red films increased *Pn*, *Gs*, *Ci*, and *Tr*, whereas green film resulted in the lowest photosynthetic activity, consistent with the absorption characteristics of chlorophyll [59–61]. Under green film, lower *Pn* and *Gs* combined with higher *Ci* indicate that non-stomatal factors limited photosynthesis [62]. Enhanced enzyme activities (Rubisco, FBPase, GAPDH, ATP synthase) under blue and red films further supported carbon assimilation and photosynthetic efficiency, whereas reduced enzyme activities under green film confirmed diminished photosynthetic capacity [63]. Light response parameters further indicated differences in light acclimation [59,60]. Higher *Pn*_*max*_, *LSP*, and *LCP* under red and blue films suggest improved adaptability to high light conditions, consistent with results in *Tetrastigma hemsleyanum* [64]. In contrast, seedlings under green film exhibited reduced photosynthetic potential and weaker light-use efficiency, similar to observations in *Populus szechuanica* var. *tibetica* [30]. Chlorophyll fluorescence analysis revealed that PSII function remained largely stable across treatments, with *Fv/Fm* values close to the optimal range (0.80-0.85) [5]. Higher *ΦPSII* and *qP* under blue film indicate more efficient photochemical energy conversion and a greater proportion of open PSII reaction centers, contributing to enhanced photosynthesis. In contrast, reduced *ΦPSII* and *qP* under green film indicate decreased photochemical efficiency and limited ATP and NADPH production [2,60]. Despite lower electron transport efficiency under red film, relatively high *Pn* suggests compensatory regulation of carbon fixation processes [55,61].

Carbohydrate accumulation reflects source-sink coordination and carbon metabolism [65]. Blue film increased glucose and fructose levels in leaves and stems, as well as sucrose accumulation in roots, indicating enhanced carbon assimilation and allocation to sink organs [66]. Similar responses have been reported in relation to blue light-regulated sucrose metabolism [67]. Red film promoted sugar accumulation in stems and roots, suggesting enhanced carbon translocation and sink development, consistent with findings in *Lactuca sativa* and *Fragaria × ananassa* [68,69]. In contrast, green film resulted in generally lower sugar contents, indicating reduced carbon assimilation and sink activity [70]. To integrate these responses, MFCE analysis was applied to evaluate overall *Sorbus tianschanica* Rupr. seedling performance [71]. Blue film treatment resulted in the highest comprehensive growth index, reflecting coordinated improvements in morphology, leaf structure, chloroplast development, photosynthetic capacity, enzyme activity, and carbohydrate accumulation. These findings demonstrate that light quality regulates seedling growth through integrated effects on structural and physiological processes, with blue light providing the most favorable conditions for *Sorbus tianschanica* Rupr. seedling development.

## Conclusions

Light quality differentially influenced the growth and photosynthetic characteristics of *Sorbus tianschanica* seedlings. Among the treatments, the blue film exerted the strongest effects, as evidenced by increased stomatal aperture, greater leaf thickness, and improved chloroplast ultrastructural integrity. These structural changes were accompanied by higher photosynthetic pigment contents, enhanced activities of key photosynthetic enzymes, and increased carbohydrate accumulation. Together, these responses improved light-use efficiency and carbon assimilation, thereby promoting seedling growth. The multilevel fuzzy comprehensive evaluation (MFCE), which integrated morphological, anatomical, physiological, and biochemical indicators, further confirmed that the blue film treatment achieved the highest overall performance among all treatments. These findings suggest that increasing the proportion of blue light, or using blue film coverings, can effectively promote early seedling development. Future studies should incorporate transcriptomic and metabolomic approaches to elucidate how different light qualities regulate the expression of key genes associated with seedling growth, thereby providing deeper mechanistic insights. In addition, isotope tracing techniques (e.g., ^13^C labeling) are recommended to clarify source-sink relationships and to verify assimilate transport and biomass allocation under different light quality conditions. Together, these approaches will help to further elucidate the mechanisms by which light quality regulates seedling growth and enhance the applicability of these findings in forestry and ecological restoration.

## Supporting information

S1 Data Spreadsheet 1. Dataset of the experiments. (XLSX)
